# EnzyACT: A Novel Deep Learning Method to Predict the Impacts of Single and Multiple Mutations on Enzyme Activity

**DOI:** 10.1021/acs.jcim.4c00920

**Published:** 2024-07-22

**Authors:** Gen Li, Ning Zhang, Xiaowen Dai, Long Fan

**Affiliations:** † Production and R&D Center I of LSS, GenScript (Shanghai) Biotech Co.,Ltd., Shanghai 200131, China; ‡ Production and R&D Center I of LSS, GenScript Biotech Corporation, Nanjing 211122, China

## Abstract

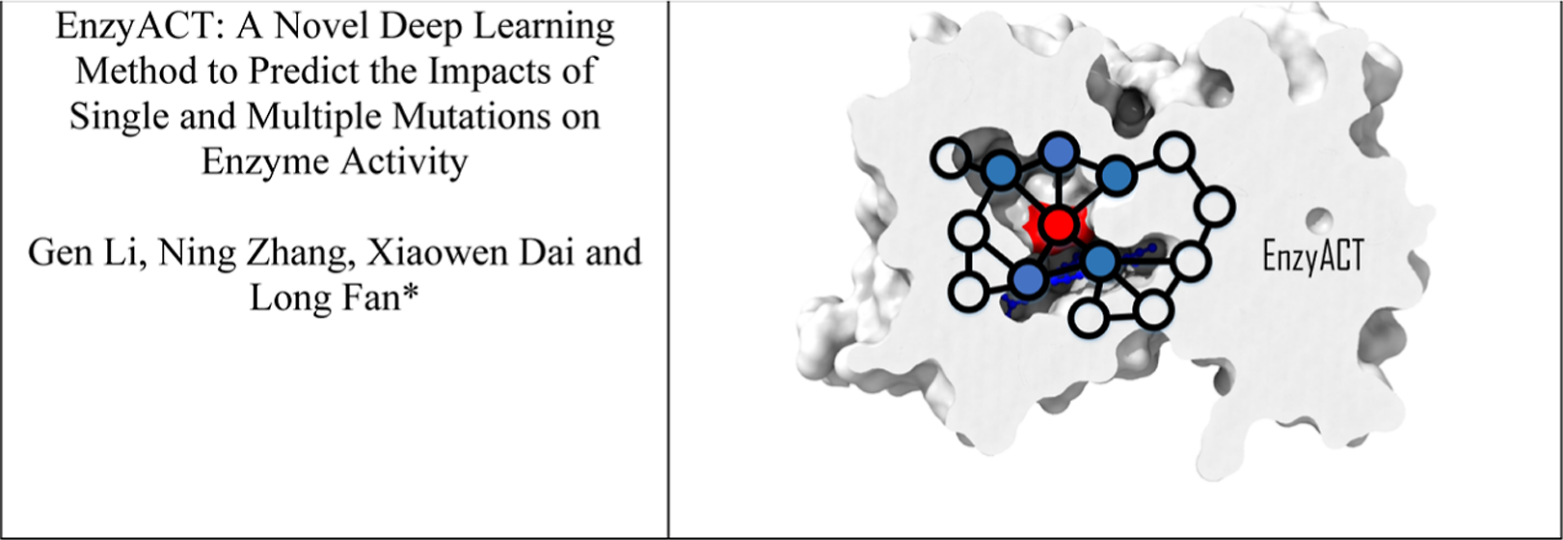

Enzyme engineering involves the customization of enzymes by introducing mutations to expand the application scope of natural enzymes. One limitation of that is the complex interaction between two key properties, activity and stability, where the enhancement of one often leads to the reduction of the other, also called the trade-off mechanism. Although dozens of methods that predict the change of protein stability upon mutations have been developed, the prediction of the effect on activity is still in its early stage. Therefore, developing a fast and accurate method to predict the impact of the mutations on enzyme activity is helpful for enzyme design and understanding of the trade-off mechanism. Here, we introduce a novel approach, EnzyACT, a deep learning method that fuses graph technique and protein embedding to predict activity changes upon single or multiple mutations. Our model combines graph-based techniques and language models to predict the activity changes. Moreover, EnzyACT is trained on a new curated data set including both single- and multiple-point mutations. When benchmarked on multiple independent data sets, it shows uniform performance on problems affected by mutations. This work also provides insights into the impact of distant mutations within activity design, which could also be useful for predicting catalytic residues and developing improved enzyme-engineering strategies.

## 1 Introduction

Most enzymes are proteins that act as catalysts in biological reactions, which play a central role in cellular activities by participating in almost every aspect of cellular function, including catalyzing decomposition,^1^ synthesizing protein,^2^ and regulating gene expression.^3^ In industrial biocatalysts, designing more efficient enzymes will help to expand the range of applications for enzymes, especially in the production of pharmaceutical intermediates.

Directed evolution and rational design are two widely used enzyme-engineering strategies. Directed evolution uses mutagenesis iterations to generate the natural evolution of mutant libraries, followed by screening for enzyme variants with desired properties. This approach requires a high-throughput (HTP) screening method, which is labor cost and time-consuming. However, rational design emphasizes the understanding of protein structure and physicochemical properties of residue interactions at the beginning of the process. It enhances our basic understanding of enzyme catalytic mechanisms, thus increasing the success of enzyme-engineering efforts and laying the foundation for functional prediction of enzymes.^4^ However, despite these successful cases, the difference in the catalytic mechanism of the protein results in a far less-extensive range of applications than directed evolution. Besides rational design and directed evolution approaches, machine learning methods have been increasingly used to find patterns in data that help predict protein properties and guide protein design.^5^

Deep learning algorithms adopt a different approach from directed evolution and rational design. In the mutation effect prediction, it utilizes the sequence, structure, physical force field, and evolutionary information^6,7^ to find new types. In recent years, deep learning has been widely used in the prediction of enzyme structure,^8^ commission numbers,^9^ substrates,^10^ and stability.^7^ In particular, dozens of deep machine learning based methods have been developed for stability prediction with impressive performance.^11^ There have been some works exploring the prediction of enzyme activity, including MutCompute,^12^ ECNet,^13^ SCANNER,^14^ UniKP,^15^ DLTKcat,^16^ and CmpdEnzymPred.^17^ SCANEER is a predicted method that uses sequence coevolution to analyze the effect of single-point mutation on enzyme activity, and limited to the algorithm, only about 47% of the variants could be evaluated. ECNet and eUniRep are deep learning methods, which provide a framework and require an activity assay of the target protein as a training set. MutCompute is a 3D-CNN model, which allows the model to learn the evolutionary information on amino acids by training on protein structures. UniKP, DLTKcat, and CmpdEnzymPred are based on machine learning or deep learning methods but they require small substrate molecule structures to predict enzyme activity. Nevertheless, there is a lack of a general machine learning- or deep learning-based method for predicting the effect of mutations on enzyme activity. Therefore, developing a fast and accurate computational method that can predict enzyme activity is a crucial step to designing customized proteins for protein engineering, personalized medicine, and precision diagnostics.

To deal with the challenge, we developed a novel approach: EnzyACT, which is a deep learning-based method that fuses structure and sequence embedding to predict enzyme activity changes upon single- or multiple-point mutations. Our model combines graph-based techniques and language models to predict activity changes. The advantage of using language model-based feature vectors is that it does not require the knowledge of catalytic mechanisms to encode the sequences.^18^ For the graph, we propose a spatial node feature to capture the residue interaction properties near mutations and use the protein sequence embedding layer of the protein language pretrained model as the spatial node feature input to Graph Convolution Network (GCN). Also, to help solve the bottleneck of developing a model limited by the data shortage, we trained EnzyACT on the training set we collected from multiple sources. It is shown to consistently outperform existing state-of-the-art methods on mutation-affected enzyme activity problems as benchmarked on several independent data sets. We provide P450 as an example where we correctly identify binding sites and catalytic sites that are known and give insights into the trade-off mechanism.

## 2 Materials and Methods

### 2.1 Data Set Preparation for EnzyACT Model Development

The data set used for our model construction was extracted from the D3DistalMutation databases^19^ on 1 Feb 2023 by customized Python scripts. We compiled a new curated data set including the protein identifier (UniProt ID), EC number, sequence, and effect of mutations (labels). As different data points usually use various synonyms to describe the effect of mutations on activity, such as increase, loss, 70% activity, and inactive, we used a customized script to ensure that the same canonical labels could be output for various synonyms, which is essential to help unify descriptive labels from different databases. These mutations with known labels satisfied the following rules: (1) nonredundant data. (2) Remove neutral mutations. (3) Remove the data with opposite labels. Several times of data washing were performed to ensure quality; 5279 single mutations decreased activity, and 220 single mutations increased activity. To balance the number of decreased activity mutations and increased activity mutations, we did the same way as protein thermostability prediction to satisfy the antisymmetric property and this data augmentation technology has been shown to improve the model robustness and performance;^11^ to put it simply, there are two types of our training data: forward, which is the original data, and inverse, which is the hypothetical inversion mutation of each forward data (the mutation type and the impact on activity are opposite). Finally, 10998 entries in 1303 proteins formed the high-quality data set for deep learning model construction, and this data set is termed S10998. Detailed information for the data set can be found in Table S1 and Figure S1.

For predicting the effects of multiple-point mutations, we removed duplicates and redundant entries (at multiple-point mutation level) from BRENDA database.^20^ To prevent overfitting due to the presence of mutations corresponding to the same or homologue proteins in both a training set and test set, we generated a more stringent test by randomly selecting about 30% of the training set as a test set and assuring that each test case has a sequence identity score less than 32% to other proteins in the training set. This led to training set M576 (233 proteins).

### 2.2 Independent Data Set for Model Testing

We compiled a series of test data sets screened from three sources: BRENDA database^20^ and papers.^21−23^ The cleaning rules for the raw data are consistent with those for the training set.

For single-point mutations, the data set used to test the performance of our model contains 6103 entries in total, of which the numbers of mutation samples that decreased activity and increased activity are 4950 and 1153, respectively. We divided the 6103 entries into S2814 and P450 to verify the performance of our model:

S2814. A blind test set contained only experimentally determined effects. It consists of 2814 single mutations in 420 proteins with experimental effects from the source as mentioned above, and 1662 of them were able to be compared with SCANEER.

P450 data set. This is a deep mutational scanning (DMS) data set of the cytochrome P450 (CYP2C9) enzyme, which contains information about changes in activity caused by mutations. DMS is an HTP technique that investigates the functionality of variants by subjecting them to a functional selection; it favors variants with high function while eliminating those with low function.^21^ Amorosi et al.^21^ employed DMS to assess the enzyme activity of numerous missense variants in P450. A total of 3289 single mutations were used for testing our model, and 1797 of them were able to be compared with those in SCANEER.

For data with multiple-point mutations, our blind test set comprises 167 entries (117 decreasing and 50 increasing; Table S2 and Figure S2), which shares low-homology (less than 32% sequence identify score) with the training set. This led to the blind set M167 (95 proteins).

In this study, wild and mutant structures were generated by using AlphaFold2^8^ with its default pipeline. Both wild-type and mutant structures were utilized for generating the features for direct and inverse mutations. All data used in this study are freely available in the Supporting Information.

### 2.3 Model for Single-Point Mutation Prediction

A GCN was created by the DGL framework,^24,25^ where the node features are derived from the ProtT5-XL-Uniref50^26^ pretrained model embedding and the spatial adjacency matrix is derived from the connection network within 10 Å near the mutation site. Finally, knowledge-based features are added to the fully connected layer. The detailed protocol and pseudocode of our model development can be found in the Supporting Information.

### 2.4 Model for Multiple-Point Mutation Prediction

We used the implementation of the Support Vector Classification (SVC) algorithm for the prediction of activity changes upon multiple-point mutations. Two classes of features were used for model training: predicted results for each single-point mutation and complementary features. This means that the output of the single-point prediction model is also used as the feature to train the model. The experimental activity data for each mutation in the training set and test sets are used to evaluate the accuracy of the combined method. Detailed information about the input features of the model can be found in Table S3.

### 2.5 Molecular Dynamics Simulation Protocol

The P450 (UniProtID: P11712) structure was downloaded from the Alphafold protein structure database,^27^ and the mutant was constructed with the Pymol Mutagenesis Wizard based on the wild type. 0.5 μs all-atom MD simulations were carried out using NAMD software with Amber ff19SB force field. The initial structures of wild type and mutant were solvated in a rectangle box with a layer of water at least 10.0 Å from the protein surface. Sufficient Na^+^ or Cl^–^ was added to neutralize the system. The simulation process follows the standard MD protocol, which consists of relaxation, heating, equilibrium, and production. The detailed steps and parameters can be found in the Supporting Information.

### 2.6 MD Trajectory Analysis

Root-mean-square deviation (RMSD), folding free energy, and dynamics cross-correlation map (DCCM) were analyzed under the AmberTools21 suite. RMSD was calculated for the protein backbone using the initial structure as the reference. The last 375 ns MD trajectory was used to calculate the folding free energy by using the MMGBSA and MMPBSA method. The DCCM was a matrix representation that could display time-related information between protein residues, which we used it for analyzing the last 375 ns MD trajectory to capture each residue motion.

## 3 Result

### 3.1 Designing a Deep Learning Framework for Activity Prediction

**Figure 1:**
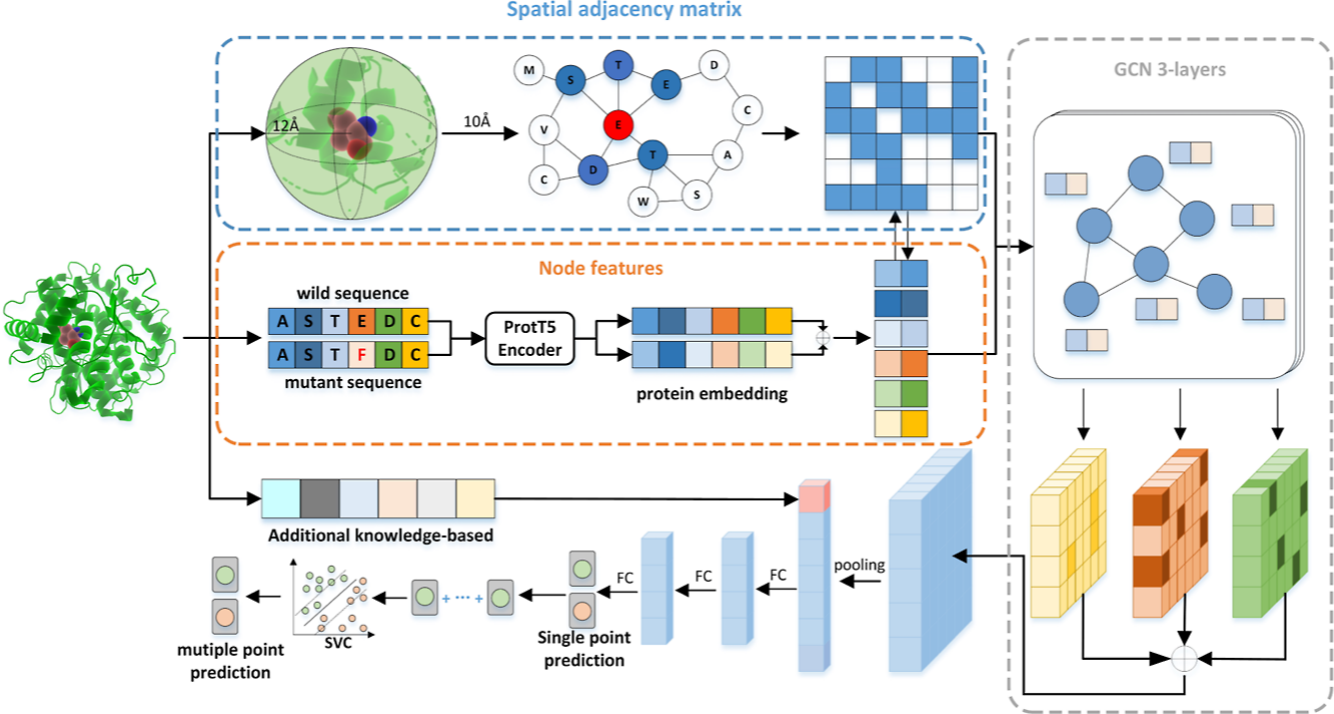
Overview of the model architecture. EnzyACT extracts two parts of information from the protein 3D structure: interaction network and sequence embedding. The protein embedding was taken from ProtT5-XL-Uniref50, each square represented a 1024-dimensional feature, and then a 2048-dimensional feature was obtained after concatenating wild and mutant. The interaction network is composed of those residues that were less than 12 Å away from the mutation site. Finally, the activity change upon mutation is predicted by the GCN model.

EnzyACT was developed by combining GCN and the SVC model for the prediction of single-point and multiple-point mutation, respectively. For the single-point prediction model, GCN was used to capture short-range residue interactions of mutation sites, while a language model was used to represent the long-range protein sequence information. For the multiple-point prediction model, a shallow machine learning method was used to prevent overfitting. The EnzyACT development workflow is summarized in Figure 1.

### 3.2 Validation of EnzyACT

We trained EnzyACT on the two largest available data sets (S10998 and M576, respectively, and defined in Methods and Materials) containing experimental data for single- and multiple-point mutations in protein enzymes, which were taken from BRENDA and D3DistalMutation. The original single-point training set includes 5279 single mutations with decreased activity and 220 single mutations with increased activity. To balance the number of decreased activity mutations and increased activity mutations, we did the same way as protein thermostability prediction to satisfy the antisymmetric property and this data augmentation technology has been shown to improve the model robustness and performance,^11^ which lead to our training set that includes 10998 samples for single point. We did not do the same thing for the multipoint training data set. Table 1 shows that the AUCs predicted with expected/experimental values are 0.98 (with an accuracy of 0.95) and 0.83 (with an accuracy of 0.83) for single- or multiple-point mutations when performed balanced fivefold validation of the data set. To evaluate whether the results of direct and inverse mutation predictions are symmetric, we defined a bias score to test the performance and our antisymmetry property is satisfied perfectly, with a bias score of just −0.01. In addition, we explored the gap between balanced and unbalanced data sets. For the single-point prediction model, the balanced data set showed better performance than the unbalanced data set (Tables S14 and S16), which means that the reverse data helped the model learn new knowledge. However, the multipoint prediction model showed the opposite result, which may be due to the uncertainty caused by the small amount of original data (Tables S8–S11). In addition, we tested some shallow machine learning methods on the balanced data set, showing that our graph-based method achieved the best results (Table S15).

**Table 1:** Performance of Prediction Methods on Fivefold Cross Validation

model	accuracy	precision	recall	F1	AUC
single	0.95	0.96	0.95	0.95	0.98
multiple	0.83	0.82	0.78	0.80	0.83

### 3.3 Predicting the Effects of Single-Point Mutations

Although several methods claim to be able to predict enzyme activity, most of them have problems that block our comparison (Table S13). To assess the ability of EnzyACT to predict the effect of single-point mutations on enzyme activity, we designed an extensive series of comparative experiments with the only available method, SCANEER, which is an MSA-based method that scans the molecular evolution of protein sequences to engineer enzyme activity.^14^ However, if SCANEER is used for predicting the effect of a mutation on activity, the position must satisfy: (1) the number of gaps is less than 20% and (2) the coevolution score is in the top N (N = protein sequence length × 2). These requirements limited its use, resulting in only about 47% of mutations that can predict changes in activity, which greatly limits the selection of engineering regions, especially when other properties need to be considered.

#### 3.3.1 Comparison of EnzyACT with the Only Existing Method on the S2814 Blind Data Set

In this case, we designed a comparable subset1 and incomparable subset2 of the S2814 test sets for comparison and independent testing, respectively. Subset1 represents the part of data set S2814 that can be predicted by SCANEER, while subset2 is the part that cannot be predicted by SCANEER.

For comparable data sets, Table 2 shows the better performance of EnzyACT in predicting enzyme mutations compared with SCANEER. As for incomparable data sets, our approach shows basically consistent performance. Finally, on the complete data set, EnzyACT obtained an accuracy of 0.70 and an AUC of 0.69 with a recall and precision of 0.12 and 0.77, respectively. In addition, we removed sequences with an identity higher than 25% from the training set for testing the generalization ability of the model. Likewise, EnzyACT outperformed the SCANEER method. We tested the performance of different secondary structures using the same method and the results showed the same accuracy (Table S4). This suggests that EnzyACT provides a consistent prediction of different enzyme activity changes and the potential for enzyme engineering.

**Table 2:** Comparative Performance of EnzyACT on the S2814 Blind Dataset^a^

data set	method	accuracy	precision	recall	F1	AUC
subset1	EnzyACT	0.71	0.80	0.13	0.23	0.68
	SCANEER	0.71	0.78	0.11	0.19	0.66
subset2	EnzyACT	0.69	0.71	0.09	0.16	0.69
all	EnzyACT	0.70	0.77	0.12	0.20	0.69
subset1_25%	EnzyACT	0.72	0.80	0.12	0.21	0.69
	SCANEER	0.71	0.79	0.10	0.18	0.66
subset2_25%	EnzyACT	0.70	0.70	0.09	0.16	0.72
all_25%	EnzyACT	0.71	0.76	0.11	0.19	0.71

a Subset1 is the subset of S2814 that can be predicted by SCANEER, while Subset2 is the rest of S2814.

#### 3.3.2 Performance on DMS Data Set for a Certain Protein Saturation Mutation

Cytochrome P450 enzymes are heme-containing proteins associated with cellular membranes that play a crucial role in the metabolism of key physiological substances across various species, including microorganisms, plants, and animals. In mammals, CYP450 enzymes are adept at identifying and processing a wide range of foreign substances including pharmaceuticals, environmental chemicals, and contaminants. Numerous isoforms of these enzymes are responsible for the oxidation and breakdown of over 90% of medications that are presently used in clinical settings.^28^

EnzyACT was further tested against the P450 data set,^21^ which is composed of experimentally determined effects of mutations in the cytochrome P450 enzyme. The mutants were subjected to deep mutational scans^29^ to calculate the activity changes of the single-point mutations. Similar to the way we processed the S2814 data set, we also designed subsets 1 and subset2. The highest sequence identity of P450 is less than 16% with our training set.

Table 3 displays a comparison of predictions in terms of AUC, accuracy precision, and recall obtained by using SCANEER predictors. EnzyACT outperforms SCANEER in terms of all metrics, and the trend is similar to the S2814 data set. This suggests that EnzyACT provides a consistent prediction of the different data sets and potential for protein engineering.

**Table 3:** Performance Comparison of EnzyACT with the SCANEER Method on P450 Datasets^a^

data set	method	accuracy	precision	recall	F1	AUC
subset1	EnzyACT	0.96	0.70	0.09	0.17	0.73
	SCANEER	0.96	0.50	0.05	0.10	0.73
subset2	EnzyACT	0.90	0.76	0.15	0.25	0.82
all	EnzyACT	0.93	0.74	0.13	0.23	0.80

a Subset1 is the subset of P450 that can be predicted by SCANEER, while subset2 is the rest of the P450 data set.

**Figure 2:**
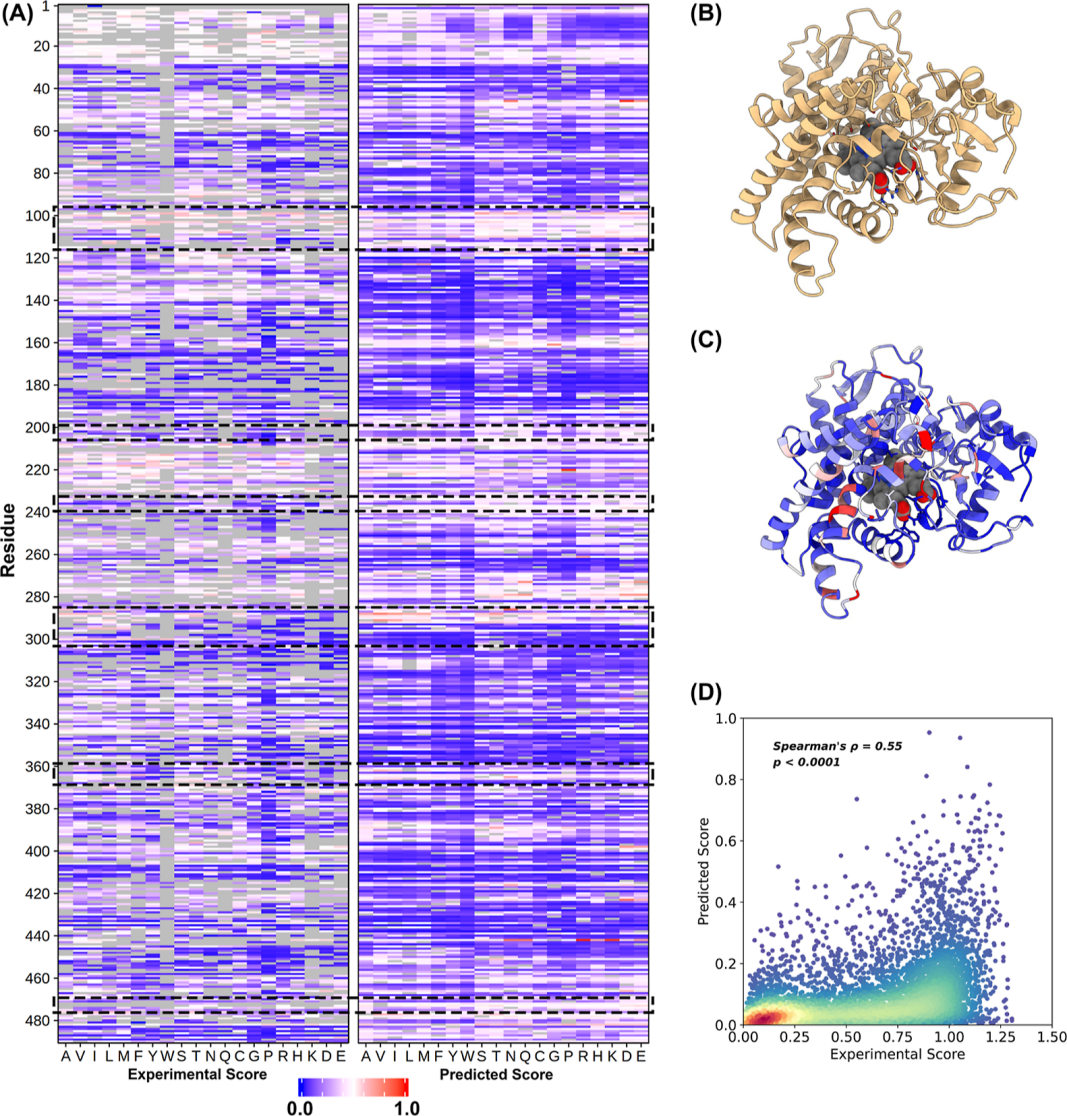
Deep analysis of P450 (CYP2C9) saturation mutation results. (A) Heatmaps of experimental activity scores (left) and predicted activity (right) scores. The missing data in experimental scores are shown in gray. Scores range from activity lost (blue) to increased (red). Active site residues are highlighted by black dashed boxes. (B) P450 (CYP2C9) complex structure (PDB: 1OG2) (C) colored by a predicted activity average score of 19 mutations. (D) Density scatterplot of activity scores between predicted and experimental.

We comprehensively analyzed the saturation mutation results of P450 to demonstrate the capability of our method to design enzyme activity. There are six key substrate regions in the P450 enzyme (Figure 2B) which are located at residues 96–117, 198–205, 233–240, 286–304, 359–369, and 470–477, all of them were highlighted by the dashed black boxes.^30^Figure 2A is the heatmap of the DMS experimental score and our predicted score, respectively, which show a clear similar pattern of activity changes upon single-point mutations, especially in these key areas. Ordinarily, substitution close to the substrate most likely changes the enzyme activity. Our results share a phenomenon similar to the experiment in that sites with increased activity that are easier to find near the pockets. The results prove not only that our method can correctly predict the impact of these key residues but also that our model can distinguish these key residue regions without using any substrate-related information, which shows the power of protein embedding.

Interestingly, we observed that the residues that can improve activity mutations not only exist in pockets but also on the surface when we mapped the data to the three-dimensional structure. This phenomenon is the same as the previous discovery by Yu and Dalby : the flexibility of different regions of the enzyme is highly correlated over long distances. Specifically, one mutation site could influence the dynamics of regions far away from it.^31^

**Figure 3:**
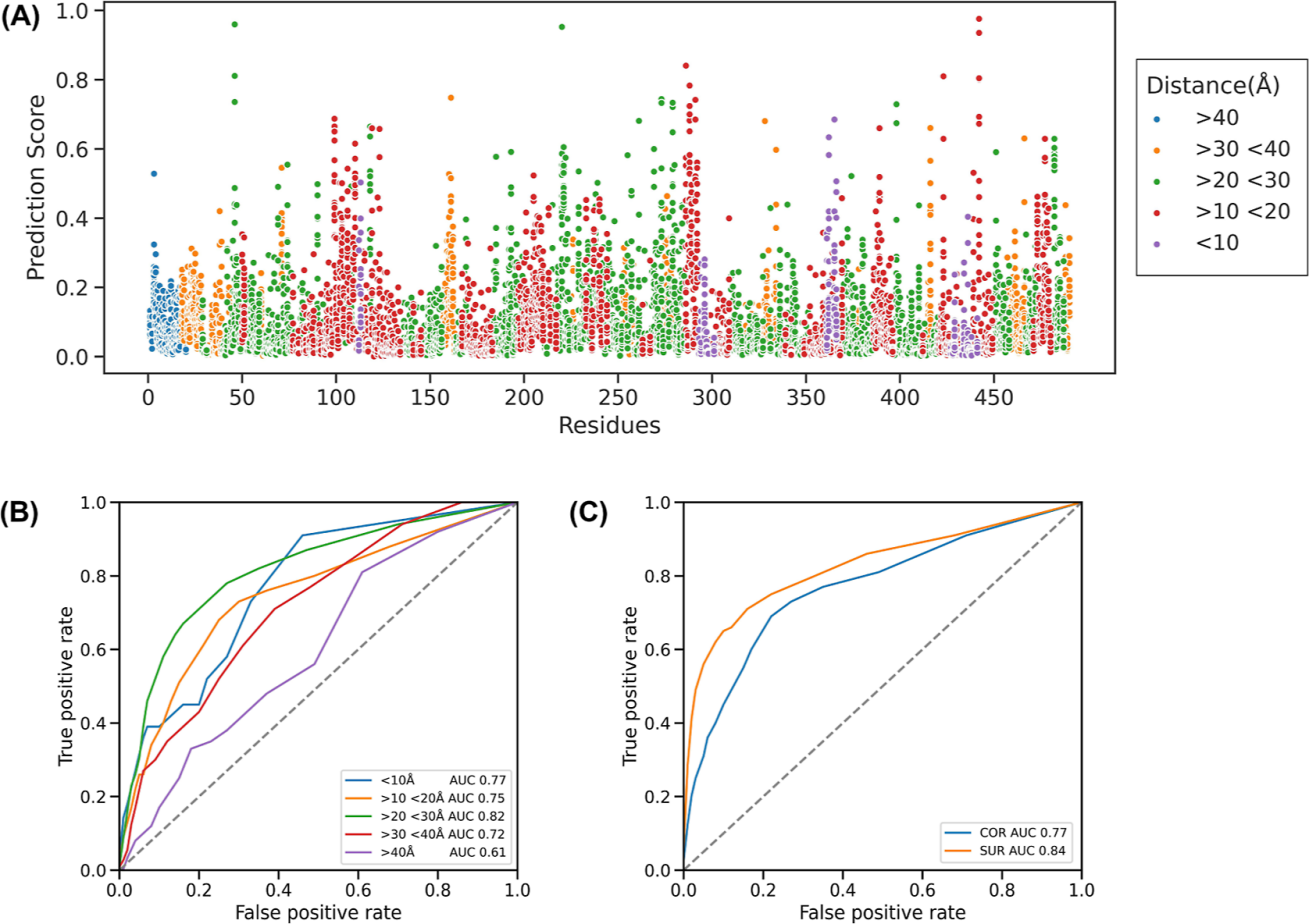
Performance over classification at different distances. (A) The prediction score for 19 variants and residue position of P450, colored by distance, which is between the weight center of an amino acid and the weight center of the small molecule. ROC curve of distance (B) and mutation position (C), which show the ability to identify activity changes on the P450 enzyme.

To further verify whether mutations far away from the pocket affect activity, we plotted a scatterplot of distance versus activity score for each residue. Of the 9310 total variants, 131 had an increased activity score and a majority of them had decreased activity. 4.7% of variants are <10 Å away from the pocket, 10 Å < 33.9% variants <20 Å, 20 Å < 45.3% variants <30 Å, 30 Å < 13.3% variants <40 Å and 3.7% variants >40 Å. As shown in Figure 3, most variants will cause a decrease in activity, which matches the theory of evolution. There are still a small number of variants that can enhance enzyme activity, not just near the pocket but even in areas larger than 30 Å. It indicates that a mutation may affect far away sites through long-range interactions and may also help to engineer the enzyme’s surface while improving enzyme activity, e.g., optimizing surface charge, enhancing stability, and improving solubility. The prediction capabilities of our method at different distances and surface/core also provide the chance for this engineering (Figure 3B,C).

Finally, we found that our prediction and experimental activity scores were well correlated (Spearman’s ρ = 0.55 *p* < 0.0001, Figure 2D). Also, we tested the performance of different secondary structures using the same method and the results showed the same accuracy (Table S5). In summary, the results demonstrate the ability of our method to identify P450 key residues, discover their novel potential active sites, and understand the catalytic mechanism.

#### 3.3.3 Cracking the Trade-Off Mechanism for P450

**Figure 4:**
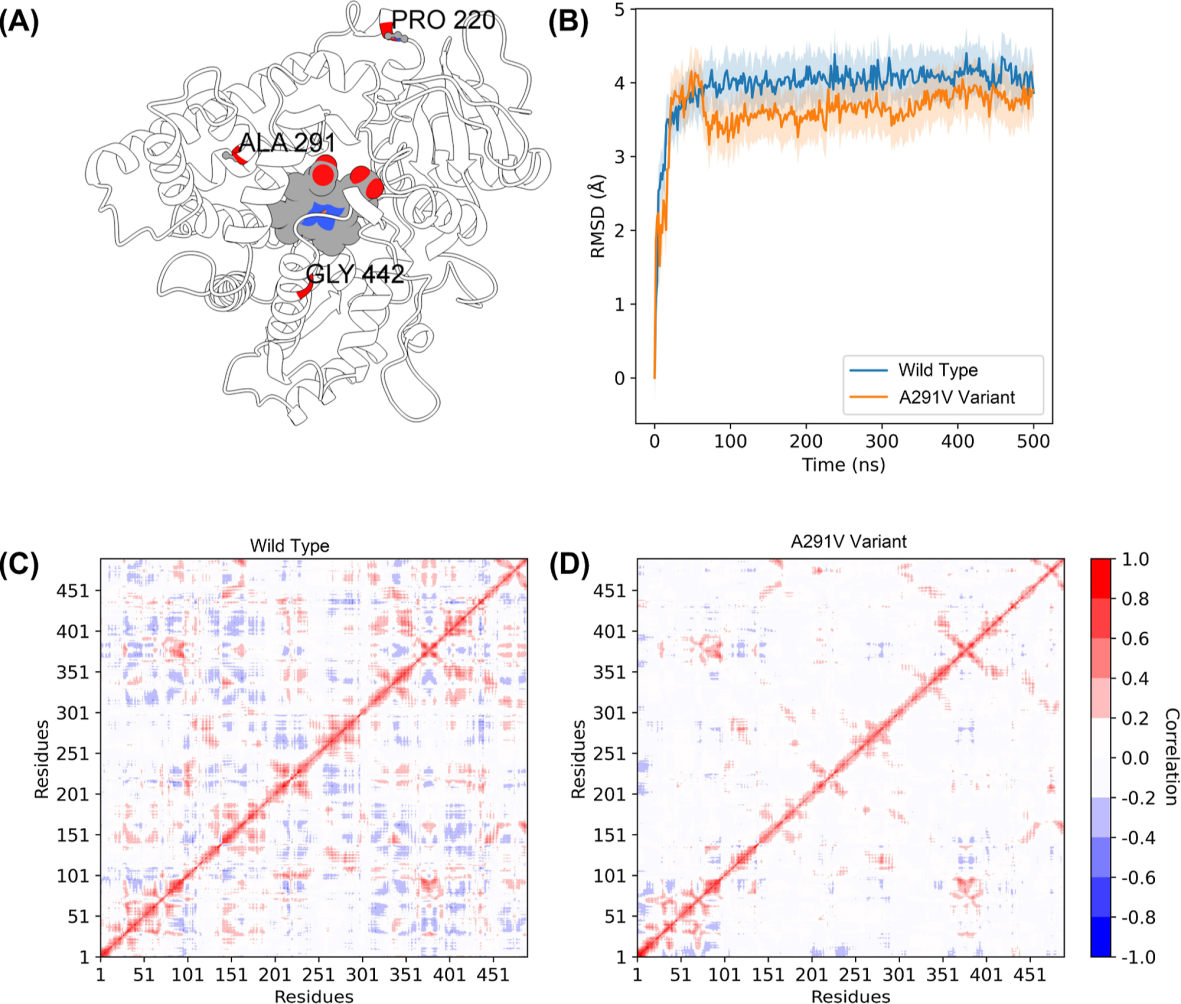
MD simulation analysis of WT and A291 V variants. (A) The mutation sites with both improved activity and improved stability were labeled on the CYP2C9 structure. (B) Comparison of the RMSD of WT and the A291 V variant during the 0.5 μs MD simulations at 298 K. (C,D) DCCM for the Cα- Cα atom pairs of the P450 WT and A291 V variant. Red color indicates two atoms moving in the same direction, i.e., positive correlation, while blue indicates the atoms moving in opposite directions, i.e., anticorrelation.

Enzymes are subject to evolutionary pressures that can lead to trade-offs between the catalytic efficiency and stability. The concept of an enzyme trade-off mechanism is important in the context of biological systems, particularly in understanding how enzymes evolve and function under different environmental conditions or pressures. Prior to this, we need to deeply understand the mechanism of the target enzyme and receive activity and stability data for each site through HTP approaches, which is unrealistic. Here, we combined EnzyACT with an advanced stability prediction method^32^ to explore the trade-off mechanism for the P450 enzyme. As a result, 131 variants had increased activity scores, but only 5 variants showed an increase in stability in a total of 9310 variants. This resulted in only four predicted variants with both improved activity and improved stability, which are S220P, A291I, A291 V, and G442R. A291I, A291 V, and G442R mutations are 10–20 Å away and S220P is 20–30 Å away from the pocket, while there is no satisfied site located near or far away from the catalytic core (Figure 4A). Typically, regions smaller than 10 Å are active sites. These regions are highly flexible but increasing stability requires increasing rigidity, which leads to the risk of losing activity. This is the so-called trade-off mechanism. Far than 30 Å is less likely to affect enzyme activity because it is far away from the active region. It indicates that the region between the active site and the outermost layer has dynamics related to the flexible active site and would provide an ideal target for enzyme engineering.^31,33^

To test this hypothesis, we performed 0.5 μs MD simulations to capture the dynamic interaction of wild type and the A291 V variant at 298 K. We chose the A291 V variant for analysis because its activity-increased effect has been experimentally confirmed.^21^ RMSD is used to indicate the stability of the protein that can be determined by the deviations produced during its simulation. In Figure 4B, the A291 V variant has significantly minor RMSD values compared to the WT, which shows that the structure of the A291 V variant is more stable. Also, MMPB/GBSA results confirm this conclusion: MMGBSA and MMPBSA are 4.25 and 30.40 kcal/mol, respectively (positive value indicates more stability). After that, we used a DCCM further to analyze the changes in the protein movement to explore whether the A291 V variant has an impact on the overall network of P450 proteins, especially the remote sites we are interested in. DCCM analysis is a helpful method for analyzing the time-dependent motions of all Cα atom pairs and reflecting the dynamic motion between residues.^34^ Surprisingly, the A291 V mutation eliminates most of the small fluctuations (<−0.4 correlation <0.4, Figure 4C,D), which means that the A291 V mutation stabilized the entire structure while retaining some of the dynamic interactions, such as residues: 1–100, 60–100, and 350–400. These retention interactions may be critical for enzyme function.

The MD results show that the A291 V variant improves both the P450 enzyme activity and stability, which proves the potential of the EnzyACT method in enzyme engineering. There are only a few sites that meet the optimization requirements of two attributes at the same time. One possible reason is that P450 has reached the state that is most suitable for the environment after long-term evolution. Another reason may be that we missed many positive samples due to the low recall of the two methods. Finally, our result demonstrates the potential of EnzyACT to crack the trade-off mechanism of P450 combined with advanced stability prediction methods.^35−37^

#### 3.3.4 Ablation Study of EnzyACT

We used features from different sources to train EnzyACT. To evaluate the relative contribution of the protein embedding compared with the others, such as spatial adjacency matrix and additional knowledge-based features, we conducted a feature ablation study by excluding protein embedding features or other features from the full-fledged EnzyACT feature set. Table S6 displays the validation performance of the ablated variants of EnzyACT in terms of the ROC-AUC values for enzyme activity prediction. All the subsets are essential for the final model. Also, we have evaluated node features ranging in size from 10 to 2048 to ascertain if larger node sizes, particularly 2048, pose a risk of overfitting. The findings, as detailed in Figure S3, alleviate concerns about overfitting and show the capability of T5 protein embeddings to effectively encapsulating information. In addition, we explored other types of graph convolutional networks previously proposed in the deep learning paper. Specifically, we tested the performance of EnzyACT on all of the data sets with GraphConv, SAGEConv, GINConv, GATConv, ChebConv, and MultiGraphConv, resulting in GraphConv achieving the best performance (Table S7).

### 3.4 Predicting the Effects of Multiple-Point Mutations

Prediction of multiple-point mutations is also an essential way for enzyme engineering to design novel enzymes. We trained a model based on the prediction of single-point mutations, which is designed in a way that is easily extended to predict activity upon multiple-site variants. Due to the lack of comparable methods that can predict activity changes upon multiple-point mutations, we evaluated our approach on a blind test set and compared it with the baseline. On the blind test set M167, EnzyACT achieved an AUC of 0.73 (Table 4). The baseline1 model performs much worse; the precision and AUC only achieve 0.17 and 0.49, respectively. Although the performance of the baseline2 model has improved compared with the baseline1 model, it still maintains low performance. Similarly, we transform SCANEER into a multipoint prediction method according to baselines 1 and 2, and the results are even worse than the untrained baseline method. The results of baselines 1 and 2 illustrate that due to the complicated interplay between residues, the overall effect cannot be predicted by simply combining multiple single-point mutations.

**Table 4:** Performance of EnzyACT on M167 Blind Datasets^a^

data set	method	accuracy	precision	Recall	F1	AUC
All	EnzyACT	0.80	0.73	0.54	0.62	0.73
	baseline1	0.68	0.17	0.02	0.04	0.49
	baseline2	0.71	0.53	0.18	0.27	0.56
subset1	EnzyACT	0.74	0.38	0.25	0.30	0.57
	SCANEER_base1	0.76	0	0	0	0.49
	SCANEER_base2	0.76	0	0	0	0.49
	baseline1	0.74	0	0	0	0.48
	baseline2	0.76	0.33	0.08	0.13	0.52
subset2	EnzyACT	0.83	0.83	0.63	0.72	0.78
	baseline1	0.65	0.25	0.03	0.05	0.49
	baseline2	0.68	0.57	0.21	0.31	0.57

a The baseline1 approach regards multiple-point mutations as consisting of multiple single-point mutations and averages all probability of output by the single-point mutation model, while baseline2 uses the method of Montanucci et al.^37^ to get the prediction (*P*^mutiple^ = *P*_max_^single^ + *P*_min_^single^ – P^single^_mean_). Since the SCANEER cannot predict the effects of multiple-point mutations, we converted SCANEER into a multipoint method using a similar approach as baselines1 and 2. Subset1 is a subset of M167 that can be predicted by SCANEER, while subset2 is the rest of the M167 data set.

Overall, EnzyACT was able to correctly classify 80% of the multiple-point mutations in the blind test set. As expected, however, across our low-homology test set, EnzyACT shows a better performance for the model trained on the direct data set, achieving precision = 0.73 (on M167), while for the model trained on the symmetrical data set, the performance drops to precision = 0.43 (on M167, Tables S8–S11). These results indicate a need for new experimental data on multiple-point mutations, especially those with activity-increasing effects, since the use of hypothetical inverse mutations is likely to add more uncertainty to the model. Reassuringly, however, our final model demonstrated balanced predictive performance across both increasing and decreasing mutations, achieving an overall accuracy of 80% and precisions of 73 and 82% on mutations that increased and decreased activity, respectively. In summary, our method has proven to be robust when tested on the low-identified and diverse mutation data set (Table S12).

## 4 Discussion

Activity and stability are two inherent properties of enzymes, but the biotechnological applications of enzymes are limited due to the existence of a trade-off mechanism^33,38^ between activity and stability, which means that an increase in activity is accompanied by a decrease in protein stability.^39^ Nevertheless, genetically and chemically modified enzymes are beginning to show that the activity–stability trade-off can be overcome.^31,33,38^ In this context, developing a fast and accurate method that can predict the activity changes upon mutations is critical for the protein-engineering community. Here, we present EnzyACT, a GCN utilizing direct and inverse mutations to account for model antisymmetry and integrates protein embedding and spatial adjacency matrix to better capture short-range residue interactions and long-range protein language information. EnzyACT uses a large training set to avoid the risk of overfitting and outperforms another predictor on various blind test sets, especially in both precision and recall. Our method can predict single- and multiple-point mutations, which combines with currently prevalent protein stability predictors to better understand the trade-off mechanism of the enzyme. We use P450 enzymes as an example to demonstrate the potential of EnzyACT in predicting activity and utilize it to decipher the trade-off mechanism. It also greatly enhances our basic understanding of enzyme-binding and catalytic mechanisms, thus increasing the success of enzyme-engineering efforts and laying the foundation for the functional prediction of new protein sequences. However, more case studies are needed in the future to verify the generalizability of the method. Moreover, our method exhibits remarkable robustness and performance resilience by attaining high predictive accuracy even when using AlphaFold2-predicted structures as input, thereby dramatically enhancing the scalability of protein activity prediction without compromising on accuracy. Also, we are developing an excellent user-friendly web server for a wide range of academic and industrial users to provide a simple and convenient way to access EnzyACT.

## Data Availability

All the data sets and results can be found in https://github.com/GenScript-IBDPE/EnzyACT

## Supporting Information Available

Data set statistics of training set and blind sets, additional knowledge-based features used in the model, model training detailed, performance for node features of different sizes, performance of different secondary structures between the two methods on the S2841 data set, ablation study of EnzyACT, validation of the model, and pseudocode of EnzyACT (PDF)
