# Supplementary material for: EnzyACT: A Novel Deep Learning Method to Predict the Impacts of Single and Multiple Mutations on Enzyme Activity
Source: J Chem Inf Model. 2024 Jul 22;64(15):5912–21. doi: 10.1021/acs.jcim.4c00920 (PMC11323264; doi:10.1021/acs.jcim.4c00920)
Supplement: Supplementary file 1 — ci4c00920_si_001.pdf [file ci4c00920_si_001.pdf]

EnzyACT: A Deep Learning Method to Predict the Impacts of Single and Multiple Mutations on Enzyme Activity

Gen Li[a]†, Ning Zhang[b]†, Xiaowen Dai[b], Long Fan[a]*

[a]Production and R&D Center I of LSS, GenScript (Shanghai) Biotech Co.,Ltd., Shanghai, 200131, China

[b]Production and R&D Center I of LSS, GenScript Biotech Corporation, Nanjing, 211122, China

†These authors contributed equally to this work.

*To whom correspondence should be addressed. Email: leo.fan@genscript.com

Development of single-point mutation models

Materials and Methods

Table S1. Training set and test set used for model development.

Dataset	All	Increased	Decreased	Proteins Involved
Training set (S5499)	5499	220	5279	1303
Test set (S2814)	2814	913	1901	420
Test set (P450)	3289	240	3049	1

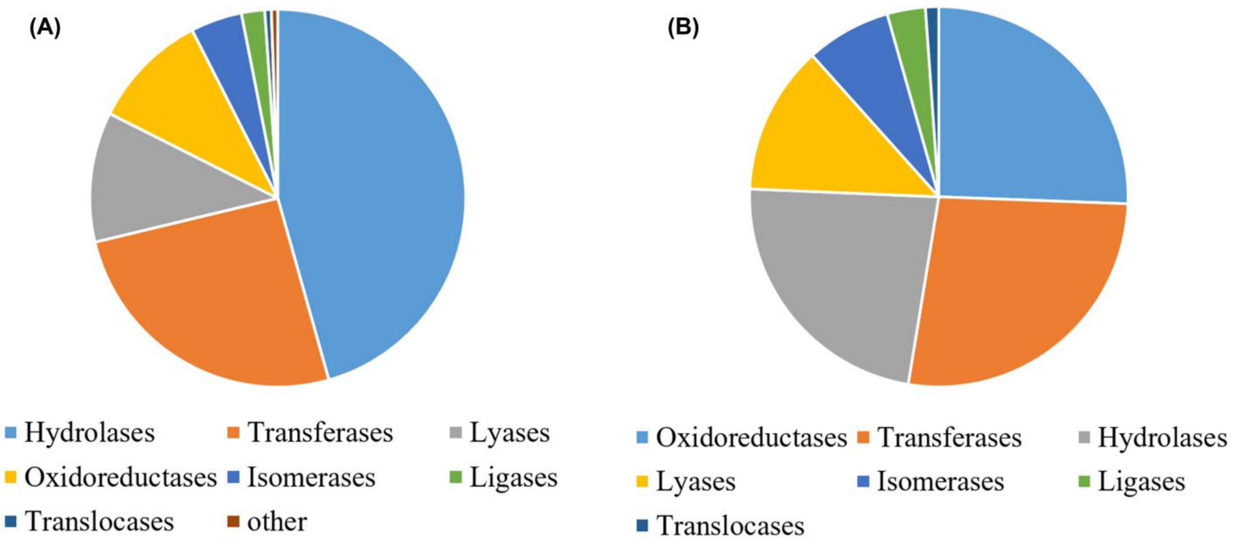

Figure S1. Ratio of different enzyme types in (A) training set and (B) test set (S2814).

Graph Convolution Network Architecture

In the protein, the nodes represent the amino acids of which proteins are made up of, and the interactions between residues make up the adjacency matrix. In this work, we use the class of dgl.nn.pytorch.conv.GraphConv in DGL (version 1.1.0)[1,2] to develop our GCN model.

Node features

Graph-based protein node features usually adopt one-hot to encode the characteristic of each node, which can be physicochemical properties or evolutionary information. In this work, we applied the protein sequence embeddings generated by the ProtT5-XL-Uniref50 pretrained model as node features. ProtT5-XL-Uniref50 pretrained model was developed by Ahmed et al.[3], which was trained on 450M protein sequences by using the T5 architecture with 3B parameters. It achieves state-of-the-art results in multiple downstream tasks compared with other popular protein language models[4]. The traditional way to obtain the node features is based on the sequence, and selected several amino acids from both the left and right of the mutation site as nodes[5]. Obviously, it is more reasonable to construct nodes through space because mutations change the surrounding interactions. One mutation is represented as the concatenating of wild sequence embedding and that of mutant sequence. This means, the node feature is a matrix of N*2048, where N is the set of residues less than 12 Å away from the mutation site and F^0=2048 is the twice size of hidden layers. We denote the matrix as mutational embedding which represents the mutational effect on the whole sequence.

Spatial adjacency matrix (SAM)

Adjacency matrix indicates whether pairs of vertices are adjacent or not in the graph. This theory is based on the protein structure as a network, in which each amino acid is directly connected according to a certain relationship, the connection between each node and other nodes is the interaction between an amino acid and other surrounding amino acids. For geometric models, we defined the Cα atom of each residue as a node, and edges were drawn between nodes if they were within 10 Å from each other. Mutational embedding corresponding to each residue was then assigned to the respective node on the protein graph.

Additional knowledge-based features (AKB)

A set of additional knowledge regarding the environmental characteristics of the wild-type residue (e.g., relative solvent accessibility, residue depth and secondary structure) was added to fully connected layers.

We use 10 additional features: PSSM score[6], ΔCS[7], *ΔOMH*[8], $SASA_{pro}$[9], $SASA_{sol}$[10], P_{FWY}, P_{RKDE}[11], P_L, N_{Hydro} and N_{Charg}[12].

Training details

The Graph Convolutional Network architectures of EnzyACT used the protein embeddings, spatial adjacency matrix and additional knowledge-based features as the input to train the model. The model utilizes the NVIDIA A100 for training, which is built using the PyTorch deep learning framework and involves extensive hyperparameter tuning. In the 3-layer graph convolutional network (GCN) model, the output dimension of each layer is 256. In the 2-layer fully connected network, the output dimension of each layer is 256. The activation function is ReLU. The model is trained for 100 epochs using an Adam optimizer with a learning rate 1e-5 and a batch size of 128. All hyperparameters are determined through a grid search based on the model's performance on the validation set.

Symmetry test method

To evaluate whether the results of direct and reverse mutation predictions are symmetric, we defined a bias score in reference to stability prediction methods:

$$\delta = \frac{\sum_{i=1}^{N}(|Direct_{label} + Reverse_{label}|)}{N}$$

Assume that the result of increased activity is 1, then the result of decreased activity is -1, a perfectly antisymmetric and unbiased method should have δ equal to 0.

Molecular Dynamics Simulation Protocol

The MD simulations of the constructed systems were performed by using the NAMD software package[13] with AMBER ff19SB force field[14]. Both the wild-type and mutant were embedded in a box-shaped (95 × 101 × 92 Å^3) bath of water molecules, and there was a layer of TIP3P water 10 Å in each direction from

the atom with the largest coordinate in that direction. The system was neutralized with sodium cations or chloride anions. Na+ and Cl− ion pairs were then added to reach a physiological salt concentration of 0.15 M. Approximately 82,300 atoms were used for each system. The solvated protein was equilibrated by carrying out a series of 4,000 steps of energy minimization with 10 kcal/mol/Å^2 restraints on the backbone, after 4,000 steps of minimization without restraints, 596 ps of heating restricted 2 kcal/mol/Å^2 on the backbone from 0 to 298 K, after heating, gradually decrease the restricted force within 500ps until 0, 1 ns of density equilibration with NVT followed by 500 ns of constant pressure equilibration at 298 K. The system was equilibrated using an NPT ensemble at 298 K and pressure at 1 atm (1 atm = 101.3 kPa). All the simulations were run with SHAKE on hydrogen atoms, a 2 fs time step and a Langevin thermostat for temperature control and pressure control. Periodic boundary conditions and the Particle-Mesh-Ewald (PME)[15] algorithm were adopted to compute the long range electrostatic forces, and the cutoff was set as 10 Å. Trajectory frames were collected at every 8 ps for a total of 500 ns.

Development of multiple-point mutation models

Materials and Methods

Table S2. Training set and test set used for model development.

Dataset	All	Increased	Decreased	Proteins Involved
Training set (M576)	576	247	329	233
Test set (M167)	167	50	117	95

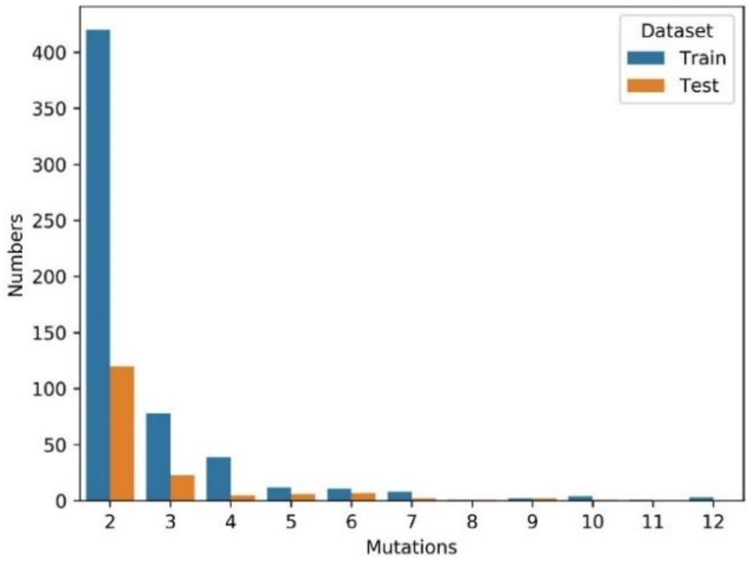

Figure S2. Distribution of mutation number in training set and test set.

Table S3. Features used in the multiple point mutation prediction

Feature	Dimension	Description
Individual Activity change	3	Max, minimum, and mean value of activity change upon each single-point mutation predicted by model
AAIndex2	92	Scores from substitution tables
Mutation distance	1	Mean value of the 3D distance between all mutation sites
Physicochemical Properties	28	Molecular Weight, Gravy, Instability, Isoelectric point, Length, Num of Second structure Helix/ Turn/Sheet, Amino_acids_content

Table S4. The performance of different secondary structures between the two methods on S2841 dataset

Dataset	Method	Secondary structure	ACC	Precision	Recall	F1	AUC
S2814 Subset1	EnzyACT	Helix	0.73	0.84	0.18	0.29	0.70
		Sheet	0.72	0.67	0.08	0.14	0.66
		Turn	0.73	1.00	0.09	0.16	0.67
		Coil	0.68	0.77	0.14	0.23	0.70
	SCANEER	Helix	0.70	0.75	0.12	0.20	0.68
		Sheet	0.74	0.82	0.11	0.20	0.62
		Turn	0.72	0.80	0.07	0.13	0.60
		Coil	0.68	0.78	0.11	0.19	0.68
S2814 Subset2	EnzyACT	Helix	0.68	0.71	0.12	0.21	0.68
		Sheet	0.79	0.60	0.07	0.12	0.72
		Turn	0.61	0.67	0.03	0.06	0.72
		Coil	0.66	0.76	0.10	0.17	0.70
S2814 All	EnzyACT	Helix	0.71	0.79	0.15	0.26	0.69
		Sheet	0.75	0.65	0.08	0.14	0.69
		Turn	0.67	0.88	0.06	0.11	0.71
		Coil	0.67	0.77	0.12	0.21	0.70

Table S5. The performance of different secondary structures between the two methods on P450 dataset

Dataset	Method	Secondary structure	ACC	Precision	Recall	F1	AUC
P450 Subset1	EnzyACT	Helix	0.94	1.00	0.11	0.19	0.70
		Sheet	0.99	NaN	0.00	0.00	0.81
		Turn	0.97	NaN	0.00	0.00	0.78
		Coil	0.98	0.25	0.10	0.14	0.80
	SCANEER	Helix	0.94	0.67	0.07	0.13	0.74
		Sheet	0.99	NaN	0.00	0.00	0.59
		Turn	0.97	0.00	0.00	0.00	0.84
		Coil	0.98	0.00	0.00	0.00	0.75
P450 Subset2	EnzyACT	Helix	0.91	0.67	0.13	0.21	0.79
		Sheet	0.90	0.00	0.00	0.00	0.90
		Turn	0.92	0.67	0.15	0.25	0.84
		Coil	0.87	0.93	0.22	0.35	0.85
P450 All	EnzyACT	Helix	0.93	0.76	0.12	0.20	0.75
		Sheet	0.95	0.00	0.00	0.00	0.91
		Turn	0.95	0.67	0.11	0.18	0.84
		Coil	0.93	0.78	0.20	0.32	0.86

“ NaN” means lack of sufficient positive data.

Table S6. Ablation study of EnzyACT

ID	Model	Features			Precision	Recall	AUC	ACC	F1
		Node features	adjacency matrix	knowledge-based					
1	Graph Convolution Network (3 layers)	T5 embedding	Spatial	Yes	0.76	0.12	0.73	0.83	0.21
2	Graph Convolution Network (3 layers)	Onehot	Spatial	Yes	0.71	0.10	0.70	0.82	0.18
3	Graph Convolution Network (3 layers)	T5 embedding	Spatial	No	0.35	0.32	0.64	0.76	0.33
4	Graph Convolution Network (3 layers)	T5 embedding	7 aa Sequence	Yes	0.64	0.13	0.70	0.82	0.21
5	Graph Convolution Network (3 layers)	T5 embedding	21 aa Sequence	Yes	0.68	0.13	0.72	0.82	0.21
6	Graph Convolution Network (1 layers)	T5 embedding	Spatial	Yes	0.72	0.10	0.68	0.82	0.18
7	Graph Convolution Network (2 layers)	T5 embedding	Spatial	Yes	0.71	0.11	0.71	0.82	0.19
8	Graph Convolution Network (4 layers)	T5 embedding	Spatial	Yes	0.64	0.15	0.70	0.82	0.24
9	FC (1 layers)	T5 embedding	No	Yes	0.70	0.13	0.69	0.82	0.22
10	FC (2 layers)	T5 embedding	No	Yes	0.69	0.13	0.67	0.82	0.22
11	FC (3 layers)	T5 embedding	No	Yes	0.67	0.15	0.64	0.83	0.24
12	FC (4 layers)	T5 embedding	No	Yes	0.66	0.15	0.67	0.82	0.24
13	FC (5 layers)	T5 embedding	No	Yes	0.66	0.16	0.62	0.83	0.26

All results are trained on S10998 and tested on S6103. “Sequence” means using the left and right N amino acids near the mutation site on the sequence to replace the amino acids near the mutation site in space (SAM), and updating the corresponding adjacent matrix. “No” means that this feature is not used for training model.

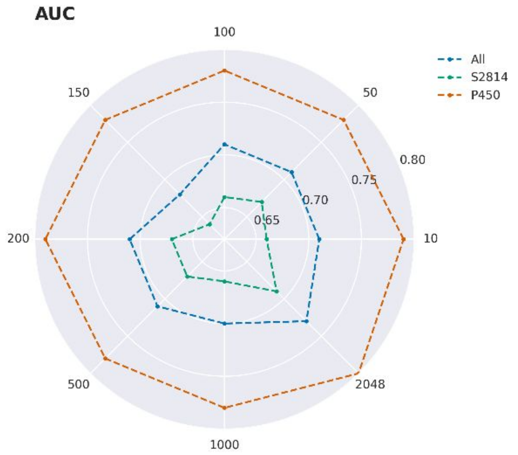

Figure S3. EnzyACT performance for node features of different sizes.

Table S7. Performance of different graph network models on the same data.

Dataset	GCN type	ACC	Precision	Recall	F1	AUC
All test	GraphConv	0.83	0.76	0.12	0.21	0.73
	SAGEConv	0.83	0.70	0.14	0.23	0.70
	GINConv	0.82	0.71	0.10	0.18	0.71
	GATConv	0.82	0.64	0.15	0.24	0.68
	ChebConv	0.82	0.71	0.12	0.20	0.70
	MultiGraphConv	0.73	0.31	0.33	0.32	0.63
S2814	GraphConv	0.70	0.77	0.12	0.20	0.69
	SAGEConv	0.70	0.70	0.12	0.21	0.68
	GINConv	0.70	0.73	0.10	0.17	0.69
	GATConv	0.70	0.72	0.13	0.23	0.70
	ChebConv	0.70	0.70	0.11	0.20	0.67
	MultiGraphConv	0.66	0.46	0.32	0.38	0.64
P450	GraphConv	0.93	0.74	0.13	0.23	0.80
	SAGEConv	0.93	0.69	0.19	0.30	0.79
	GINConv	0.93	0.65	0.13	0.21	0.77
	GATConv	0.93	0.48	0.19	0.27	0.76
	ChebConv	0.93	0.74	0.12	0.21	0.79
	MultiGraphConv	0.80	0.15	0.38	0.22	0.67

Table S8. 5-Fold Cross-validation Performance

Model-Direct	Accuracy	Precision	Recall	F1_score	AUC
LogisticRegression	0.80	0.76	0.77	0.76	0.79
SVC	0.83	0.82	0.78	0.80	0.83
DecisionTreeClassifier	0.76	0.73	0.70	0.71	0.75
RandomForestClassifier	0.85	0.86	0.77	0.81	0.84
AdaBoostClassifier	0.80	0.78	0.75	0.76	0.80
XGBClassifier	0.84	0.82	0.81	0.81	0.84
Baseline1	0.71	0.74	0.51	0.61	0.69
Baseline2	0.69	0.73	0.41	0.52	0.65

Training set: Only direct

Table S9. Test set (M167) Performance

Model-Direct	Accuracy	Precision	Recall	F1_score	AUC
LogisticRegression	0.65	0.45	0.68	0.54	0.66
SVC	0.80	0.73	0.54	0.62	0.73
DecisionTreeClassifier	0.48	0.29	0.52	0.37	0.49
RandomForestClassifier	0.68	0.45	0.36	0.40	0.59
AdaBoostClassifier	0.74	0.57	0.58	0.57	0.70
XGBClassifier	0.69	0.48	0.50	0.49	0.63
Baseline1	0.68	0.17	0.02	0.04	0.49
Baseline2	0.71	0.53	0.18	0.27	0.56
SVC (Individual activity change removed)	0.79	0.71	0.50	0.59	0.71

Training set: Only direct

Table S10. 5-Fold Cross-validation Performance

Model-Inverse	Accuracy	Precision	Recall	F1_score	AUC
LogisticRegression	0.71	0.71	0.71	0.71	0.71
SVC	0.73	0.74	0.72	0.73	0.74
DecisionTreeClassifier	0.70	0.70	0.71	0.70	0.70
RandomForestClassifier	0.76	0.77	0.75	0.76	0.76
AdaBoostClassifier	0.69	0.70	0.70	0.69	0.69
XGBClassifier	0.81	0.81	0.81	0.81	0.81
Baseline1	0.63	0.69	0.49	0.57	0.63
Baseline2	0.63	0.70	0.47	0.56	0.63

Training set : Direct+Inverse

Table S11. Test set (M167) Performance

Model-Inverse	Accuracy	Precision	Recall	F1_score	AUC
LogisticRegression	0.68	0.46	0.46	0.46	0.61
SVC	0.65	0.43	0.48	0.45	0.60
DecisionTreeClassifier	0.52	0.31	0.48	0.38	0.51
RandomForestClassifier	0.66	0.41	0.28	0.33	0.55
AdaBoostClassifier	0.64	0.37	0.28	0.32	0.54
XGBClassifier	0.74	0.58	0.44	0.50	0.65
Baseline1	0.68	0.17	0.02	0.04	0.49
Baseline2	0.71	0.53	0.18	0.27	0.56

Training set : Direct+Inverse

Table S12. Performance of the final model on datasets with different numbers of mutations

SVC - Direct	Accuracy	Precision	Recall	F1_score	AUC
CV5	0.83	0.82	0.78	0.80	0.83
Test (All kinds of mutations)	0.80	0.73	0.54	0.62	0.73
Test (2 mutations)	0.80	0.69	0.31	0.43	0.63
Test (3 mutations)	0.91	0.88	0.88	0.88	0.90
Test (4 mutations)	0.60	-	-	-	-
Test (5 mutations)	0.83	0.80	1.00	0.89	0.75
Test (over 5 mutations)	0.69	0.78	0.78	0.78	0.64

Training set: Only direct, Test set:M167.

Table S13. Information about incomparable tools for enzyme activity prediction

**Method**	**Algorithm**	**Reason**	**Doi**	**Year**
MutCompute	CNN	The website cannot be registered and no source code is provided. Emil not response.	10.1021/acssynbio.0c00345	2020
ECNet	LSTM	It only provides a framework and can only be used for specific proteins with training sets.	10.1038/s41467-021-25976-8	2021
eUniRep	RNN	It only provides a framework and can only be used for specific proteins with training sets.	10.1038/s41592-021-01100-y	2021
CmpdEnzymPred	CNN	Only determines whether the input substrate matches the enzyme and does not involve mutations.	PMID: 36530936/No Doi	2022
DLTKcat	GAN+CNN	The blind dataset only contains kcat parameters	10.1093/bib/bbad506	2023
UniKP	Extra Trees	The blind dataset only contains kcat parameters	10.1038/s41467-023-44113-1	2023
GVP-MSA	GNN+MLP	Code error	10.1016/j.cels.2023.07.003	2023

Table S14. 5-fold cross validation results for balanced and unbalanced datasets.

Dataset	ACC	Precision	Recall	F1	AUC
CV5 (Direct+Inverse)	0.95	0.96	0.95	0.95	0.98
CV5（Direct）	0.96	0.62	0.19	0.29	0.81

Table S15. 5-fold cross validation results for machine learning methods on balanced dataset.

Method	Accuracy	Precision	Recall	F1	AUC
EnzyACT	0.95	0.96	0.95	0.95	0.98
RandomForest	0.92	0.92	0.92	0.92	0.93
LogisticRegression	0.95	0.95	0.95	0.95	0.97
SVC	0.95	0.95	0.95	0.95	0.97
DecisionTree	0.89	0.89	0.89	0.89	0.89
AdaBoost	0.94	0.95	0.94	0.94	0.94
XGB	0.95	0.95	0.95	0.95	0.96

Table S16. EnzyACT trained on balanced and unbalanced data set and tested on the S2814 dataset.

Dataset	Method	Accuracy	Precision	Recall	F1	AUC
Subset1	EnzyACT (Direct+inverse)	0.71	0.80	0.13	0.23	0.68
	EnzyACT (Direct)	0.67	0.36	0.03	0.05	0.56
	SCANEER	0.71	0.78	0.11	0.19	0.66
Subset2	EnzyACT (Direct+inverse)	0.69	0.71	0.09	0.16	0.69
	EnzyACT (Direct)	0.67	0.49	0.02	0.04	0.61
All	EnzyACT (Direct+inverse)	0.70	0.77	0.12	0.20	0.69
	EnzyACT (Direct)	0.67	0.39	0.03	0.05	0.58
Subset1_25%	EnzyACT (Direct+inverse)	0.72	0.80	0.12	0.21	0.69

	EnzyACT (Direct)	0.67	0.26	0.02	0.04	0.56
	SCANEER	0.71	0.79	0.10	0.18	0.66
Subset2_25%	EnzyACT (Direct+inverse)	0.70	0.70	0.09	0.16	0.72
	EnzyACT (Direct)	0.68	0.52	0.02	0.04	0.63
All_25%	EnzyACT (Direct+inverse)	0.71	0.76	0.11	0.19	0.71
	EnzyACT (Direct)	0.68	0.34	0.02	0.04	0.59

Pseudocode of EnzyACT

using ProtT5-XL-Uniref50 model to get sequence embedding as node feature

Function read_node_feature(sequence)

  node_feature = Embedding_Extraction(sequence)

  return node_feature

End function

using sequence and structure information to get knowledge based features (AKB)

```
Function read_knowlegde_based_feature(sequence, structure)
  knowledge_based_embedding = knowledge_based_Embedding(sequence, structure)
  return knowledge_based_embedding
End function

# generate graph architecture
Function graph_generation(structure, cutoff_node=12, cutoff_edge=10, mutation, node_features)
  distance_matrix = write_distance_matrix(structure)
  result_list = []
  for i from 1 to length(distance_matrix) do
    distance = calculate_distance(distance_matrix[i-1], mutation)
    if distance ≤ cutoff_node then
      result_list.append(i)
  End for
  adjacency_matrix = generate_adjacency_matrix(distance_matrix, result_list, cutoff_edge)
  graph = write_graph(node_features, adjacency_matrix)
  return graph
End function



# ActivityClassificationModel
Function ClassificationGraphConvolution(n_conv=3, n_fcn=3, graph)
  node_features = []
  for i = 1 to n_conv:
    node_feature = GraphConv(graph, graph.node_feature)
    node_feature = relu(node_feature)
    node_features.append(h)
  End for
  node_features_mean = Pooling(concatenate(node_features))
```

```
  embedding = concatenate(node_features_mean, knowledge_based_embedding)
  for i = 1 to n_fcn:
      embedding = Linear(embedding)
      embedding = relu(embedding)
  End for
  probability = classification_linear(embedding)
  return probability
```

End function

```
#Training
dataset = Dataset(graphs, labels)
loss_function = cross_entropy
optimizer = Adam
for epoch = 1 to num_epochs:
  for batch in dataset:
      inputs, targets = batch
      predictions = model(inputs)
      loss = loss_function(predictions, targets)
      optimizer.zero_grad()
      loss.backward()
      optimizer.step()
```

Hyperparameters of SVM model

```
{'C': 1.0,
 'break_ties': False,
 'cache_size': 200,
 'class_weight': None,
 'coef0': 0.0,
 'decision_function_shape': 'ovr',
 'degree': 3,
```

'gamma': 'scale',

'kernel': 'rbf',

'max_iter': -1,

'probability': True,

'random_state': None,

'shrinking': True,

'tol': 0.001,

'verbose': False}

Reference

(1) Wang, M.; Zheng, D.; Ye, Z.; Gan, Q.; Li, M.; Song, X.; Zhou, J.; Ma, C.; Yu, L.; Gai, Y.; Xiao, T.; He, T.; Karypis, G.; Li, J.; Zhang, Z. Deep Graph Library: A Graph-Centric, Highly-Performant Package for Graph Neural Networks. arXiv August 25, 2020. http://arxiv.org/abs/1909.01315 (accessed 2023-11-17).

(2) Kipf, T. N.; Welling, M. Semi-Supervised Classification with Graph Convolutional Networks. arXiv February 22, 2017. http://arxiv.org/abs/1609.02907 (accessed 2023-11-17).

(3) Elnaggar, A.; Heinzinger, M.; Dallago, C.; Rehawi, G.; Wang, Y.; Jones, L.; Gibbs, T.; Feher, T.; Angerer, C.; Steinegger, M.; Bhowmik, D.; Rost, B. ProtTrans: Toward Understanding the Language of Life Through Self-Supervised Learning. *IEEE Trans. Pattern Anal. Mach. Intell.* **2022**, *44* (10), 7112–7127. https://doi.org/10.1109/TPAMI.2021.3095381.

(4) Unsal, S.; Atas, H.; Albayrak, M.; Turhan, K.; Acar, A. C.; Doğan, T. Learning Functional Properties of Proteins with Language Models. *Nat. Mach. Intell.* **2022**, *4* (3), 227–245. https://doi.org/10.1038/s42256-022-00457-9.

(5) Gligorijević, V.; Renfrew, P. D.; Kosciolek, T.; Leman, J. K.; Berenberg, D.; Vatanen, T.; Chandler, C.; Taylor, B. C.; Fisk, I. M.; Vlamakis, H.; Xavier, R. J.; Knight, R.; Cho, K.; Bonneau, R. Structure-Based Protein Function Prediction Using Graph Convolutional Networks. *Nat. Commun.* **2021**, *12* (1), 3168. https://doi.org/10.1038/s41467-021-23303-9.

(6) Bhagwat, M.; Aravind, L. PSI-BLAST Tutorial. In *Comparative Genomics*; Bergman, N. H., Ed.; Walker, J. M., Series Ed.; Methods in Molecular Biology; Humana Press: Totowa, NJ, 2007; Vol. 395, pp 177–186. https://doi.org/10.1007/978-1-59745-514-5_10.

(7) Choi, Y.; Sims, G. E.; Murphy, S.; Miller, J. R.; Chan, A. P. Predicting the Functional Effect of Amino Acid Substitutions and Indels. *PLoS ONE* **2012**, *7* (10), e46688. https://doi.org/10.1371/journal.pone.0046688.

(8) Sweet, R. M.; Eisenberg, D. Correlation of Sequence Hydrophobicities Measures Similarity in Three-Dimensional Protein Structure. *Journal of Molecular Biology* **1983**, *171* (4), 479–488. https://doi.org/10.1016/0022-2836(83)90041-4.

(9) Joosten, R. P.; Te Beek, T. A. H.; Krieger, E.; Hekkelman, M. L.; Hooft, R. W. W.; Schneider, R.; Sander, C.; Vriend, G. A Series of PDB Related Databases for Everyday Needs. *Nucleic Acids Research* **2011**, *39* (Database), D411–D419. https://doi.org/10.1093/nar/gkq1105.

(10) Rose, G. D.; Geselowitz, A. R.; Lesser, G. J.; Lee, R. H.; Zehfus, M. H. Hydrophobicity of Amino Acid Residues in Globular Proteins. *Science* **1985**, *229* (4716), 834–838. https://doi.org/10.1126/science.4023714.

(11) Hou, Q.; Kwasigroch, J. M.; Rooman, M.; Pucci, F. SOLart: A Structure-Based Method to Predict Protein Solubility and Aggregation. *Bioinformatics* **2020**, *36* (5), 1445–1452. https://doi.org/10.1093/bioinformatics/btz773.
(12) Yang, Y.; Urolagin, S.; Niroula, A.; Ding, X.; Shen, B.; Vihinen, M. PON-Tstab: Protein Variant Stability Predictor. Importance of Training Data Quality. *IJMS* **2018**, *19* (4), 1009. https://doi.org/10.3390/ijms19041009.
(13) Kalé, L.; Skeel, R.; Bhandarkar, M.; Brunner, R.; Gursoy, A.; Krawetz, N.; Phillips, J.; Shinozaki, A.; Varadarajan, K.; Schulten, K. NAMD2: Greater Scalability for Parallel Molecular Dynamics. *Journal of Computational Physics* **1999**, *151* (1), 283–312. https://doi.org/10.1006/jcph.1999.6201.
(14) Tian, C.; Kasavajhala, K.; Belfon, K. A. A.; Raguette, L.; Huang, H.; Migues, A. N.; Bickel, J.; Wang, Y.; Pincay, J.; Wu, Q.; Simmerling, C. ff19SB: Amino-Acid-Specific Protein Backbone Parameters Trained against Quantum Mechanics Energy Surfaces in Solution. *J. Chem. Theory Comput.* **2020**, *16* (1), 528–552. https://doi.org/10.1021/acs.jctc.9b00591.
(15) Essmann, U.; Perera, L.; Berkowitz, M. L.; Darden, T.; Lee, H.; Pedersen, L. G. A Smooth Particle Mesh Ewald Method. *The Journal of Chemical Physics* **1995**, *103* (19), 8577–8593. https://doi.org/10.1063/1.470117.
